# Outside‐In Inverted L‐Shaped Capsulotomy in Hip Arthroscopy

**DOI:** 10.1002/atn2.70378

**Published:** 2026-11-07

**Authors:** YiSen Feng, JianLong Ni, ZeNan Tian, ZhiHao Chen, Wang Wei, DaZhi Wang, ZhiBin Shi

**Affiliations:** ^1^ Department of Sports Medicine and Pediatric Orthopaedics, The Second Affiliated Hospital of Xi'an Jiaotong University Xi'an Shaanxi China

## Abstract

Hip arthroscopy has become the mainstream surgical treatment for femoroacetabular impingement syndrome, with approximately 88.75% of patients returning to sports postoperatively. Capsulotomy is a critical step in arthroscopic labral repair; however, various reported techniques possess inherent limitations. This technique employs an outside‐in longitudinal capsulotomy along the femoral neck, followed by a transverse capsular release from its acetabular attachment along the path of the labral tear, creating an inverted “L”‐shaped opening. On completion of the repair, the hip capsule is closed and retensioned using the remaining sutures from the labral repair anchors. This article summarizes the advantages, disadvantages, pearls, and pitfalls of this technique.

## Graphical abstract

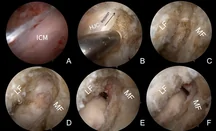

**VIDEO 1:** This technical video shows the outside‐in inverted L‐shaped capsulotomy technique in a left hip viewed from the anterolateral portal. The capsule is secured to the acetabulum using the remaining suture anchors originally placed for labral repair. This approach results in less damage to the hip capsule, particularly preserving the integrity of the iliofemoral ligament. The video illustrates patient positioning in the supine position, portal establishment, stepwise longitudinal and transverse capsulotomy, and anchor‐based capsular closure, providing a comprehensive educational presentation of the entire surgical procedure. Step 1: Under arthroscopic visualization via the anterolateral portal, first, locate the iliocapsularis muscle as an anatomical landmark for incision. Then, we use radiofrequency ablation and a shaver to remove the adipose and connective tissue overlying the hip joint capsule, taking care to preserve the capsular tissue. After clearing the surgical field, starting lateral to the iliocapsularis muscle, an outside‐in technique is employed to perform a longitudinal capsulotomy along the axis of the femoral neck. This incision provides exposure to parts of the peripheral compartment and the femoral head. Because the incision is deepened toward the reflected head of the rectus femoris, traction can be applied once the femoral head is reached. When approaching the labrum, a shaver can be used to complete the longitudinal capsulotomy. Step 2: With traction applied to the hip joint during the longitudinal capsulotomy, the central compartment is exposed. A probe is used to assess the labral pathology. Once the extent of the labral injury is identified, radiofrequency ablation is used to perform a transverse capsulotomy along the line of the labral tear, separating the capsule from its acetabular attachment, until the damaged labrum is sufficiently exposed for repair. This creates a complete inverted “L”‐shaped capsulotomy, comprising a longitudinal capsulotomy and exposing the surgical field. Step 3: After the completion of the labral repair and correction of bony deformities, a 70° suture passer is first used to penetrate the hip joint capsule, delivering a polydioxanone suture to the peripheral compartment near the remaining sutures from the labral repair. The remaining suture and the polydioxanone suture are then retrieved together using a suture grasper. After the sutures are pulled out, the passing loop is tightened to pass an absorbable suture through the joint capsule. The same procedure is repeated on the opposite side. For the longitudinal incision closure, a side‐to‐side anastomosis technique is employed, using a 70° suture passer to penetrate 1 side of the joint capsule, following the same method as used for the transverse incision closure. After all sutures have been passed through the joint capsule, they are tied to complete the closure of the capsular incision, thereby reattaching the acetabulum to the freshened acetabular bed—analogous to the tendon‐to‐bone healing process in rotator cuff repair. Another situation: In cases where the labral tear is extensive, an extended transverse capsulotomy is required; multiple full‐suture anchors reserved for labral repair can be placed with approximately 5‐mm spacing between each anchor. For the longitudinal incision, side‐to‐side repair is performed: first, a suture punch is used to pass a suture through the lateral capsular flap, whereas a suture hook is used to pass the corresponding suture through the medial capsular flap. Typically, 1 to 2 sutures are used. The remaining sutures from these anchors are passed directly through the joint capsule using the same technique described above. These sutures are then tied directly with their corresponding counterparts to achieve closure. After all sutures have been passed through the joint capsule, they are securely tied to complete the closure of the extended capsulotomy, effectively reattaching the joint capsule to the acetabulum. Video content can be viewed at https://doi.org/10.1002/atn2.70378.

Femoroacetabular impingement syndrome, recognized as early as 1936, had its pathophysiology outlined in the 2016 Warwick Agreement. It involves ongoing damage from abnormal bony‐soft‐tissue contact and is a primary cause of adult hip pain.^1^, ^2^ Diagnostic rates and hip arthroscopy volumes have risen substantially;^3^ after arthroscopy, approximately 84.4% return to sport.^4^ A recent large‐database analysis further confirmed this trend, reporting a 97% increase in hip arthroscopy cases in the United States from 2015 to 2023 (from 1236 to 2427 cases), with projections exceeding 4800 annual cases by 2030.^5^ Nevertheless, revision hip arthroscopy rates have also increased, ranging from 2% to 17%, with under‐resection of cam or pincer lesions and isolated labral debridement as primary predictors, and outcomes after revision are inferior to those of primary procedures.^6^ Biomechanical research advocates routine capsular closure to maintain stability and optimize outcomes.^7^

Current capsulotomy techniques include interportal, T‐shaped, and puncture approaches.^8^, ^9^, ^10^ The T‐capsulotomy transects the iliofemoral ligament and zona orbicularis,^11^ and the central junction often remains incompletely sealed, predisposing to microinstability.^12^ Conversely, interportal capsulotomy causes less iatrogenic damage but offers a restricted surgical field, limiting applicability.^13^, ^14^

To address these challenges, we describe a capsulotomy that maximizes the preservation of the iliofemoral ligament and zona orbicularis while providing ample visualization for labral repair and osteochondroplasty (Video 1). During closure, residual suture limbs and anchors reattach the capsule to the acetabular rim, restoring the native anatomical footprint and facilitating tendon‐to‐bone healing observed in rotator cuff repair.^15^ This ensures complete closure of the central junction and restores inherent capsular tension.

## SURGICAL TECHNIQUE

### Patient Positioning and Portal Establishment

Under general anesthesia, the patient is positioned supine on a traction table. The affected hip is flexed to 15° in neutral rotation. A perineal post is placed. The anterior superior iliac spine and greater trochanter are marked. The standard anterolateral (AL), midanterior, and distal accessory anterolateral portals are routinely marked. After standard skin preparation and draping, the procedure begins with the AL portal. A blunt obturator with a cannula is advanced through the muscular interval until resistance from the lateral hip capsule is felt, indicating entry into the extra‐articular space above the capsule. This portal serves as the primary viewing portal. A modified midanterior portal is then established 1 cm medial to the marked midanterior site. After a superficial skin incision, a switching stick or a semicannula is inserted. Under visualization from the AL portal, the instrument is brought into the field. This portal serves as the primary working portal. The initial view is superior to the hip capsule on the superficial aspect medial to the iliocapsularis muscle.

### Outside‐In Longitudinal Capsulotomy

The hip is viewed from the AL portal. Radiofrequency ablation (Johnson & Johnson, New Brunswick, NJ) and a shaver (Arthrex, Naples, FL) are first used to clear the adherent fat and connective tissue overlying the surface of the hip joint capsule, taking care to preserve the capsular tissue itself. After achieving a clear surgical field, the most lateral edge of the acetabulum is identified. The direct and reflected heads of the rectus femoris are visualized. This area is characterized by a thicker connective tissue with fibers running counter to the iliofemoral ligament, facilitating its identification. Establishing this landmark serves as a “lighthouse” for the subsequent longitudinal incision.

The longitudinal capsulotomy starts lateral to the iliocapsularis tendon (Figure 1A). Using an outside‐in technique, the incision is made along the course of the femoral neck. This exposes portions of the peripheral compartment and the femoral head. Because the incision deepens, it approaches the reflected head of the rectus femoris. Traction can be applied because the incision nears the femoral head (Figure 1B‐E). A shaver is used to carefully extend the incision toward the labrum to avoid iatrogenic injury, continuing until the labrum is fully exposed (Figure 1F).

**FIGURE 1:**
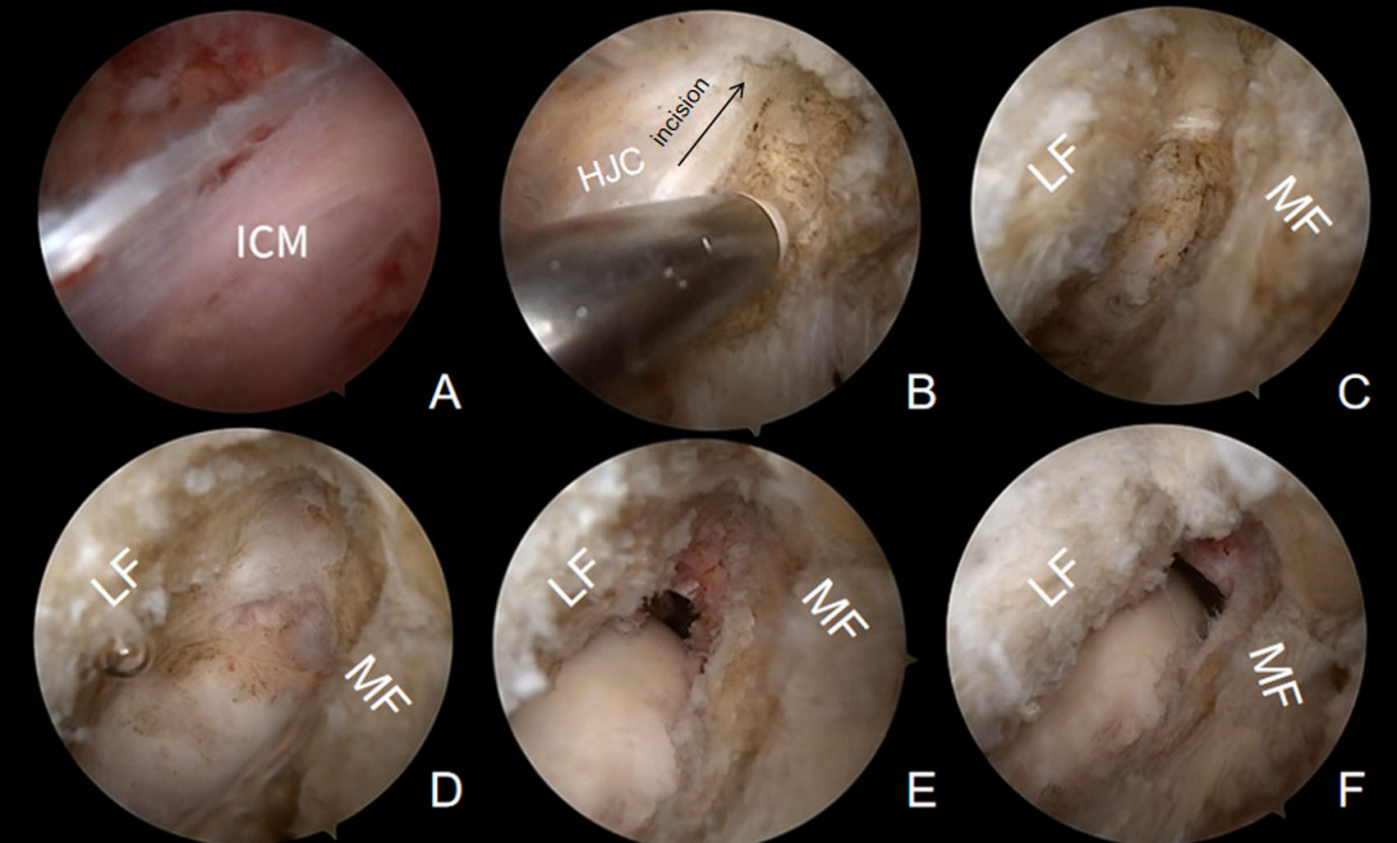
Left hip, supine position, viewed from the anterolateral portal. This series shows the outside‐in longitudinal capsulotomy. The incision is initiated just lateral to the ICM tendon (A) and progressively extended medially (B‐E) until the full thickness of the HJC is incised, creating a longitudinal opening with distinct LF and MF. This approach eliminates the need for pretraction and spinal needle localization, thereby reducing traction‐related neurovascular injuries and iatrogenic labral punctures. The complete capsulotomy affords excellent intraoperative visualization of the entire joint, facilitating subsequent labral assessment and repair while preserving the integrity of the capsular margins for later closure. The arrow indicates the direction of the capsulotomy incision. (F) The final repaired state. (HJC, hip joint capsule; ICM, iliocapsularis muscle; LF, lateral flap; MF, medial flap.)

### Transverse Capsular Release Along the Labral Tear

After the longitudinal incision, a shaver is used to debride both sides of the cut, removing residual connective tissue. Traction, already applied during the longitudinal phase, exposes the central compartment. A probe is used to assess the labral tear (Figure 2A). Radiofrequency ablation is then employed to perform a transverse release of the capsule from its acetabular attachment, following the path of the labral tear, until the torn labrum is adequately exposed. This transverse release, combined with the longitudinal incision, completes the inverted “L”‐shaped capsulotomy (Figure 2B‐F). After completing the labral repair and osseous correction, the capsule is routinely closed.

**FIGURE 2:**
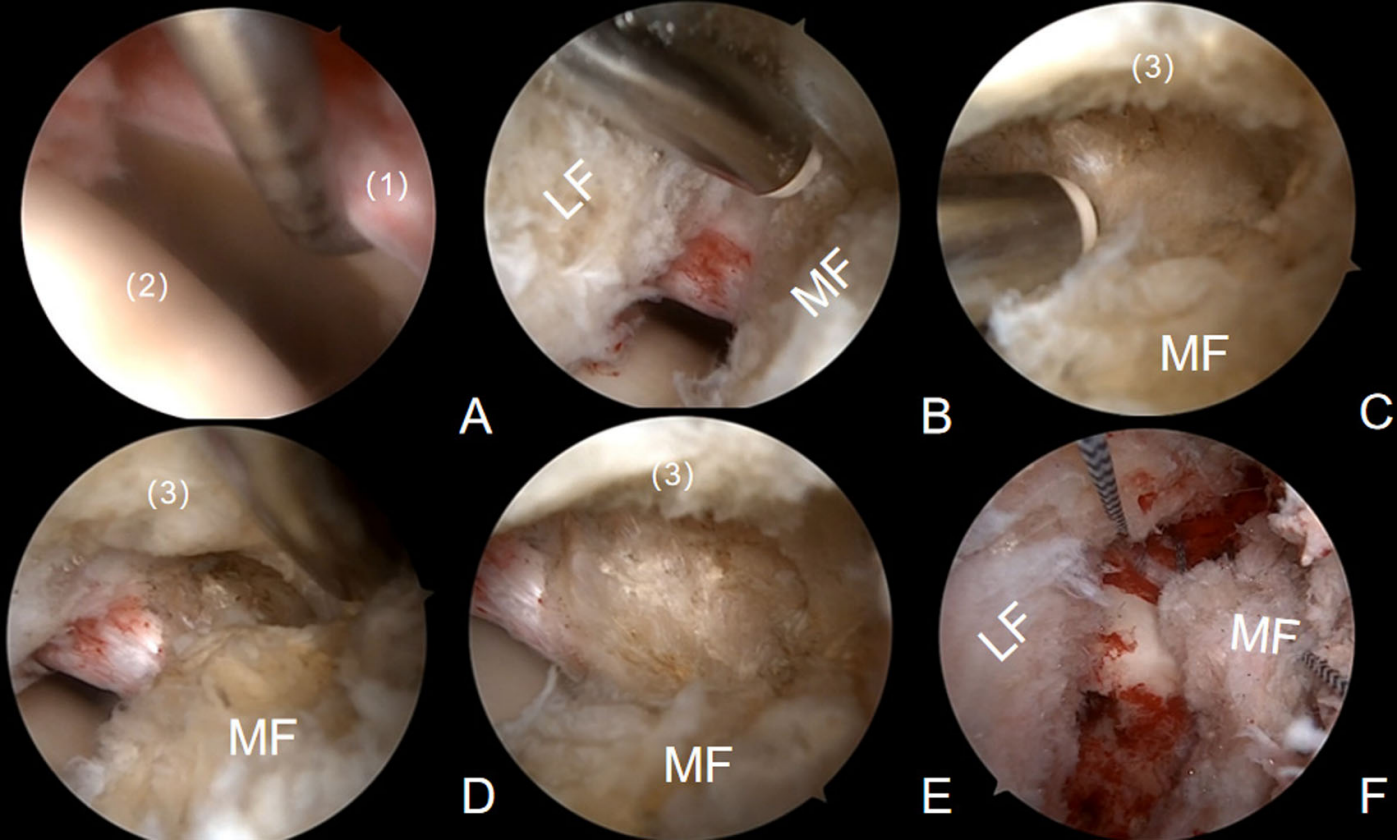
Left hip, supine position, viewed from the anterolateral portal. After completing the longitudinal capsulotomy, a medial‐sided labral tear is probed and clearly identified (A). The transverse release is then performed by extending the incision medially along the tear trajectory (B‐E), ultimately forming an inverted “L”‐shaped capsulotomy (F). Key anatomical landmarks are labeled: labrum (1), femoral head (2), and reflected head of the rectus femoris (3). This stepwise release provides unrestricted access to the labral pathology and acetabular rim, whereas the inverted L configuration preserves a robust capsular sleeve that facilitates anatomic repair and minimizes the risk of postoperative instability. (LF, lateral flap; MF, medial flap.)

### Capsular Closure

The pericapsular tissues are prepared prior to closure. First, a 70° suture passer (Rejoin Medical, Shanghai, China) is used to penetrate the hip capsule at the crossover point. A polydioxanone suture (Ethicon, Somerville, NJ) is passed into the peripheral compartment adjacent to the remaining sutures from the labral repair. A suture retriever (Rejoin Medical) is used to grasp both the remaining labral suture and the polydioxanone suture together. After retrieving the sutures, the pass‐through loop is tightened, pulling an absorbable suture through the capsule (Figure 3A). Once both suture limbs are retrieved, they are tied, closing the crossover portion of the capsulotomy and resecuring the capsule to the acetabulum (Figure 3C and D).

**FIGURE 3:**
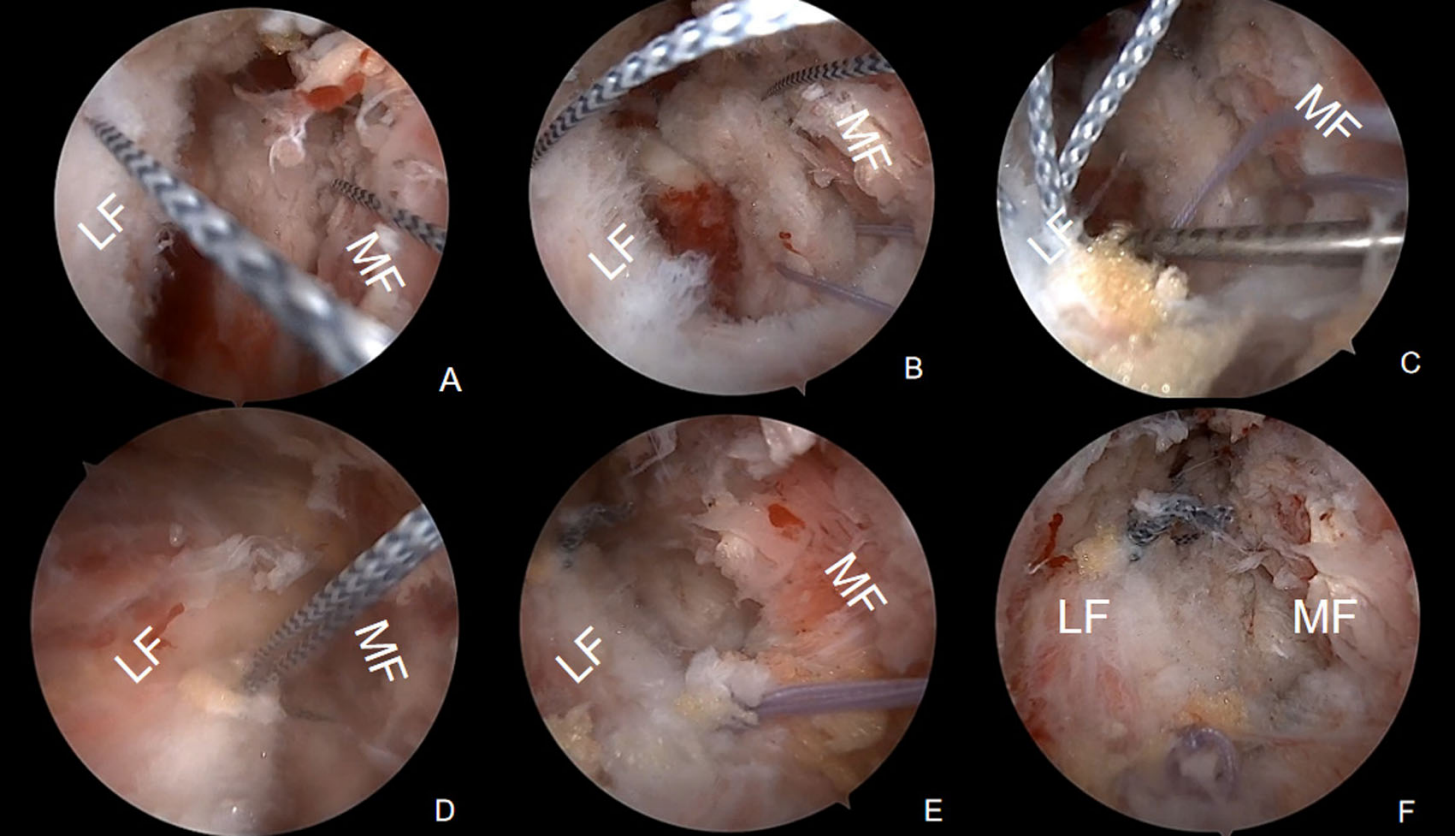
Left hip, supine position, viewed from the anterolateral portal. Capsular closure is initiated by passing a labral anchor suture through the crossover portion of the hip capsule (A), followed by a suture through the longitudinal limb (B). Sutures are then retrieved and securely tied (C‐E), resulting in a completely closed capsule that is reattached to its native anatomical footprint on the acetabulum (F). This anchor‐based fixation technique ensures strong, tensioned capsular repair, which is critical for restoring native capsular tension, reducing the risk of iatrogenic instability, and promoting optimal postoperative hip biomechanics. The LF and MF are clearly visualized throughout the closure process. (LF, lateral flap; MF, medial flap.)

The longitudinal limb is closed using a side‐to‐side repair. A 70° suture passer is used to penetrate 1 capsular flap, and the suture is passed through the capsule using the same method as for the transverse closure. The same is repeated for the opposite flap (Figure 3B). After all passes are completed, the sutures are tied to finalize the closure (Figure 3E). The longitudinal closure typically proceeds from the crossover point toward the distal end of the incision, usually requiring 1 to 2 sutures for a watertight seal. The transverse release is often short and is completely closed during the repair of the crossover point (Figure 3F).

If an extensive labral tear necessitates a larger transverse release, additional full‐suture anchors (Rejoin Medical) reserved for the labral repair can be placed with approximately 5‐mm spacing (Figure 4A and B). The sutures from these anchors are used to directly pass through the apex of the released capsular edge (using the same passing technique) and are tied to their counterparts (Figure 4C‐H). This method uses the labral repair sutures to reattach the capsule to the freshened acetabular bed. Both the medial and lateral capsular flaps can be reattached using this principle (Figure 4I).

**FIGURE 4:**
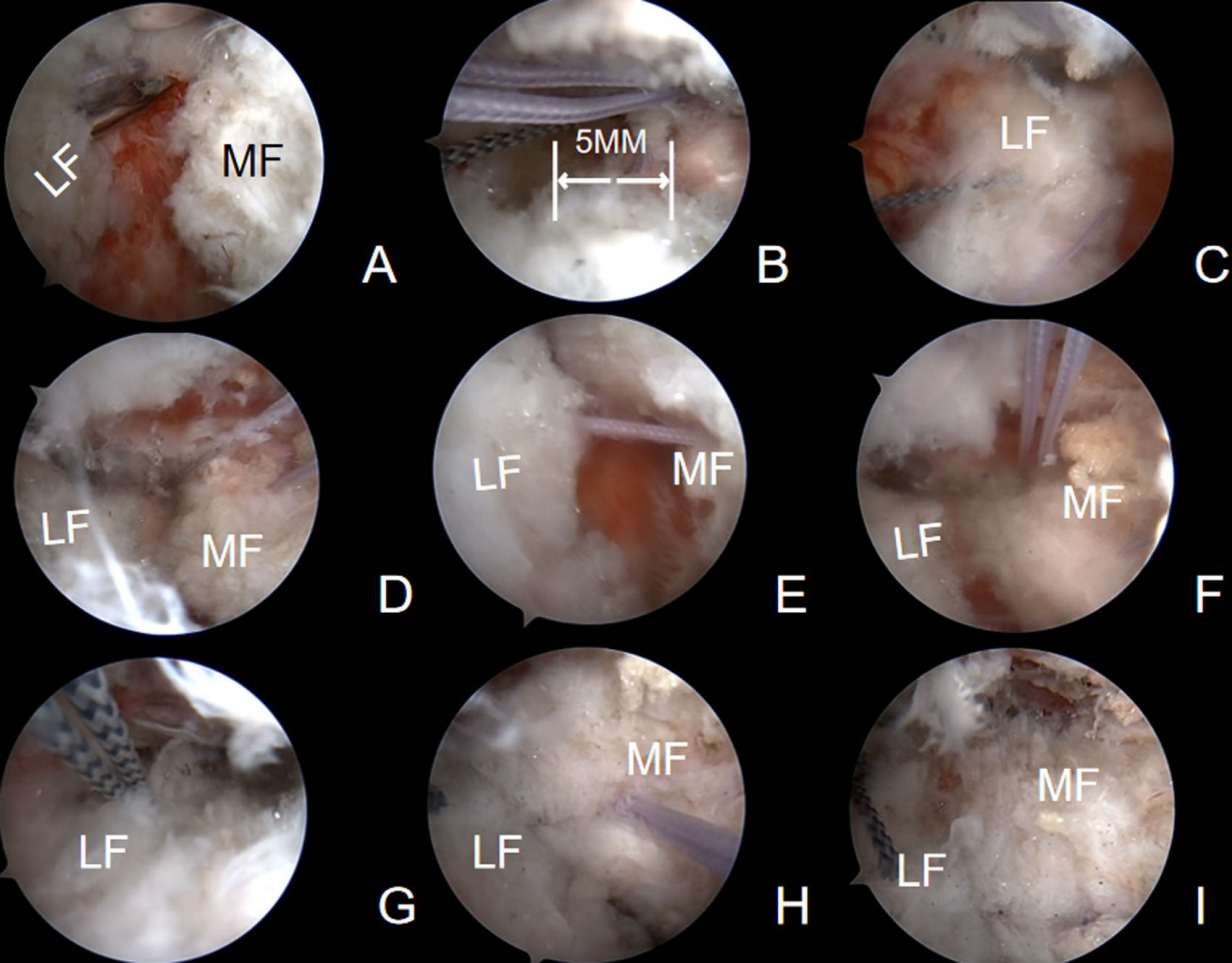
Left hip, supine position, viewed from the anterolateral portal. This sequence illustrates closure of an extended capsulotomy (larger than standard). A suture punch passes a suture through the LF (A). Two full‐suture anchors are preplaced approximately 5 mm apart on the acetabular side (B) to provide secure fixation points. The lateral suture is passed through the LF (C) and the medial suture through the MF (D). Additional sutures are passed for the longitudinal closure through both flaps (E). After suture retrieval and sequential tying (F‐H), the capsule is fully closed and reattached to its native acetabular insertion (I). For extended releases, 1 or 2 extra reserved sutures are recommended to ensure secure fixation. This technique allows complete capsular restitution even after extensive transverse release, thereby maintaining joint stability and facilitating early postoperative rehabilitation. (LF, lateral flap; MF, medial flap.)

### Postoperative Management

The portals are closed routinely postoperatively. A multimodal analgesic cocktail is administered. Rehabilitation follows a standardized protocol for femoroacetabular impingement syndrome, involving progressive hip exercises.

## DISCUSSION

In this technical note, we describe an inverted “L”‐shaped hip capsulotomy technique and summarize its advantages and disadvantages (Table 1). A growing body of evidence indicates that meticulous postoperative capsular closure, with restoration of the native anatomy and inherent tension, is critical for reconstituting the distractive stability of the hip joint. This effectively prevents postoperative microinstability and the subsequent development of secondary osteoarthritis.^16^, ^17^, ^18^ Regarding the traditional T‐capsulotomy, current closure techniques (e.g., simple sutures, figure‐of‐8 sutures, and double‐pulley circumferential augmentations) are generally complex and technically demanding.^12^, ^19^ Although Özbek et al. proposed a traction‐assisted closure protocol to simplify the procedure, repeated traction inherently increases the risk of associated traction‐related nerve injuries.^20^

**TABLE 1:** Advantages and Disadvantages of the Technique

**Advantages**	**Disadvantages**
No pretraction required, reducing traction‐related injury risk	Extra‐articular portal establishment demands high anatomical knowledge and an experienced arthroscopist
Eliminates the need for spinal needle localization, reducing iatrogenic labral puncture risk	AL portal establishment carries risk of lateral femoral cutaneous nerve injury
Simplified capsular closure, with anchor‐based fixation providing robust repair	Steeper learning curve, requiring a surgeon with substantial clinical experience
More complete capsular closure, leading to higher postoperative capsular tension	
Enhanced intraoperative visualization reduces risk of iatrogenic labral injury	

AL, anterolateral.

Our technique streamlines the capsular closure process by using the residual suture limbs from the anchors. Employing a simplified repair concept akin to the management of L‐shaped rotator cuff tears, the remaining absorbable sutures from the labral repair are used to secure the hip capsule back to its native anatomical footprint, thereby promoting robust tendon‐to‐bone healing.^15^ Although this repair strategy has a well‐established, long‐term application in rotator cuff pathology, it has rarely been described in the context of hip capsular repair.

Adequate surgical exposure is a pivotal factor in hip arthroscopy. This technique allows for the capsulotomy to be extended based on intraoperative findings while simultaneously accommodating capsular suspension. During the subsequent closure, additional suture anchors can be placed as needed, allowing the capsule to be closed using the aforementioned method.

Although traction is a necessary step in hip arthroscopy, prolonged traction times can lead to pudendal nerve neuropraxia and soft‐tissue injury.^21^ Because the present technique does not require pretraction, the overall traction time is minimized, thereby mitigating the risk of these complications.

Furthermore, this approach eliminates the need for preoperative fluoroscopically guided spinal needle localization. This streamlines the operative workflow and prevents potential iatrogenic labral injury. When capsular hypertrophy or other factors obscure the proper capsulotomy trajectory intraoperatively, a blunt trocar or radiofrequency wand can still be used for fluoroscopic orientation.

Conversely, this technique demands a thorough understanding of 3‐dimensional hip anatomy and superior spatial awareness, requiring execution by an experienced arthroscopic surgeon. Coupled with limited routine exposure and training for this specific approach, the learning curve is steep (Table 2).

**TABLE 2:** Pearls and Pitfalls of the Technique

**Pearls**	**Pitfalls**
Use a probe for blunt dissection when the capsulotomy nears articular cartilage to avoid chondral injury	Exercise caution with radiofrequency ablation near the labrum during longitudinal capsulotomy to prevent iatrogenic injury
Capsular suspension can be performed if visualization is insufficient	Take care not to confuse sutures during capsular repair because of identical suture colors
Initiate traction as the longitudinal incision reaches the femoral head to prevent accidental cutting into the labrum	An extended transverse release may require 1‐2 additional reserved sutures for secure capsular fixation
The incision can be extended as needed based on surgical requirements	

In conclusion, this technique offers an effective alternative for the surgical management of femoroacetabular impingement syndrome. The inverted L‐shaped capsulotomy shows excellent early repair outcomes. Future anatomical studies are warranted to further evaluate the associated risks of neurovascular injury. Additionally, biomechanical testing and long‐term clinical follow‐up are necessary to validate the long‐term efficacy of this technique.

## DECLARATION OF GENERATIVE AI AND AI‐ASSISTED TECHNOLOGIES IN THE WRITING PROCESS

During the preparation of this work, the authors used DeepSeek for translation. After using this tool, the authors reviewed and edited the content as needed and take full responsibility for the content of the published article.

## DISCLOSURES

The authors (Y.S.F., J.L.N., Z.N.T., Z.H.C., W.W., D.Z.W., Z.B.S.) declare that they have no known competing financial interests or personal relationships that could have appeared to influence the work reported in this article.

## References

[1] Griffin DR , Dickenson EJ , O’Donnell J , et al. The Warwick Agreement on femoroacetabular impingement syndrome (FAI syndrome): An international consensus statement. Br J Sports Med. 2016;50:1169‐1176. 10.1136/bjsports-2016-096743 27629403

[2] Johnson BT , Hunter CDR , Metz AK , Rounds AD , Singh A , Aoki SK . Impingement in patients with femoroacetabular impingement syndrome routinely occurs below 90° of hip flexion. Arthroscopy. 2026;42:125‐132. 10.1002/arj.70005 41838521

[3] Quesada‐Jimenez R , Shah PP , Strok MJ , Becker N , Kahana‐Rojkind AH , Domb BG . Hip arthroscopy yields favorable outcome, return‐to‐sport rate, and 5‐year survivorship in athletic population with generalized ligamentous laxity in the treatment of femoroacetabular impingement and labral tears. Arthroscopy. 2026;42:902‐913. 10.1002/arj.70078 41830496

[4] Migliorini F , Baroncini A , Eschweiler J , Knobe M , Tingart M , Maffulli N . Return to sport after arthroscopic surgery for femoroacetabular impingement. Surgeon. 2023;21:21‐30. 10.1016/j.surge.2021.11.006 34953722

[5] Adelstein JM , Sanghvi PA , Smith K , et al. Increased use of hip arthroscopy in the United States from 2015 to 2023 and projected growth through 2030. Arthroscopy. 2025;41:3954‐3963. 10.1016/j.arthro.2025.03.034 40147600

[6] Villarreal‐Espinosa JB , Gómez‐Verdejo F , Murray MJ , et al. Revision hip arthroscopy yields inferior patient reported outcome measures and patient acceptable symptomatic state along with higher total hip arthroplasty conversion compared to the primary setting at a minimum 2‐year follow‐up. Arthroscopy. 2025;41:5378‐5390. 10.1016/j.arthro.2025.07.014 40714224

[7] Bindi VE , Hao KA , Freeman DA , et al. Comparison of pain scores and functional outcomes of patients undergoing arthroscopic hip labral repair and concomitant capsular repair or plication versus no closure. Orthop J Sports Med. 2024;12:23259671241243303. 10.1177/23259671241243303 PMC11032060 38646603

[8] Gillinov SM , Siddiq BS , Cherian NJ , Martin SD . Puncture capsulotomy technique during hip arthroscopy. JBJS Essent Surg Tech. 2024;14:e23.00061. 10.2106/JBJS.ST.23.00061 PMC11186812 38903605

[9] Lee DR , Simon KN , Levy BA , Krych AJ , Hevesi M . Capsular management in hip arthroscopy: Interportal and T‐capsulotomy, suspension, and closure. Arthrosc Tech. 2024;13:102893. 10.1016/j.eats.2023.102893 PMC10995803 38584622

[10] Chahla J , Sherman B , Wydra F , Gerhardt MB . The pie‐crusting technique for capsular management during hip arthroscopy. Arthrosc Tech. 2019;8:e93‐e96. 10.1016/j.eats.2018.09.005 PMC6408742 30899657

[11] Lee H , Lim W , Lee S , Jo S . Impact of capsulotomy on hip biomechanics during arthroscopy. Medicina (Kaunas). 2022;58:1418. 10.3390/medicina58101418 PMC9611987 36295579

[12] Meng X , Zhao P , Wang X , Zou Z , Huang Z , Wang Z . Arthroscopic T‐capsulotomy with capsular traction and double‐pulley circumferential reinforcement for femoroacetabular impingement: A biomechanically optimized closure technique. Arthrosc Tech. 2025;14:103728. 10.1016/j.eats.2025.103728 PMC12541763 41132305

[13] Bogunovic L , Gottlieb M , Pashos G , Baca G , Clohisy JC . Why do hip arthroscopy procedures fail? Clin Orthop Relat Res. 2013;471:2523‐2529. 10.1007/s11999-013-3015-6 PMC3705062 23637056

[14] Cvetanovich GL , Levy DM , Beck EC , et al. A T‐capsulotomy provides increased hip joint visualization compared with an extended interportal capsulotomy. J Hip Preserv Surg. 2019;6:157‐163. 10.1093/jhps/hnz021 PMC6662960 31660201

[15] Bedi A , Bishop J , Keener J , et al. Rotator cuff tears. Nat Rev Dis Primers. 2024;10:8. 10.1038/s41572-024-00492-3 38332156

[16] Boos AM , Wang AS , Lamba A , et al. Long‐term outcomes of primary hip arthroscopy: Multicenter analysis at minimum 10‐year follow‐up with attention to labral and capsular management. Am J Sports Med. 2024;52:1144‐1152. 10.1177/03635465241234937 38516883

[17] Looney AM , McCann JA , Connolly PT , Comfort SM , Curley AJ , Postma WF . Routine capsular closure with hip arthroscopic surgery results in superior outcomes: A systematic review and meta‐analysis. Am J Sports Med. 2022;50:2007‐2022. 10.1177/03635465211023508 34403279

[18] Mortensen AJ , Featherall J , Metz AK , et al. The role of the hip capsule in restoring stability in the initial phase of hip distraction: An in vivo analysis. Orthop J Sports Med. 2024;12:23259671241249719. 10.1177/23259671241249719 PMC11113053 38784788

[19] Aoki SK , Karns MR , Hananouchi T , Todd DC . Hip arthroscopy capsular closure: The figure of eight technique. Arthrosc Tech. 2017;6:e505‐e509. 10.1016/j.eats.2016.10.021 PMC5443352 28580274

[20] Özbek EA , Miller L , James M , Mauro CS . Hip capsular closure in distraction: A technique to allow easier closure of T and interportal capsulotomies. Arthrosc Tech. 2023;12:e1305‐e1309. 10.1016/j.eats.2023.03.023 PMC10466195 37654878

[21] Habib A , Haldane CE , Ekhtiari S , et al. Pudendal nerve injury is a relatively common but transient complication of hip arthroscopy. Knee Surg Sports Traumatol Arthrosc. 2018;26:969‐975. 10.1007/s00167-017-4783-4 29119283

